# Deep Learning-Based Automated Detection, Segmentation, and Quantification of Retinal Fluid on Optical Coherence Tomography: A Validation Study

**DOI:** 10.3390/diagnostics16213497

**Published:** 2026-10-26

**Authors:** Nasiq Hasan, Adarsh Gadari, Sharat Chandra Vupparaboina, Shreyaa Rohindra Lall, Maria Emfietzoglou, Diana Flores-Pena, Giulia Gregori, Atharva Ajay Vichare, Utkarsh Doshi, Elham Sadeghi, Niroj Kumar Sahoo, Oscar Matteo Gagliardi, Sandeep Chandra Bollepalli, Kiran Kumar Vupparaboina, Jay Chhablani

**Affiliations:** 1 Department of Ophthalmology, University of Pittsburgh, Pittsburgh, PA 15260, USA; nasiq.imtiaz@gmail.com (N.H.); oscarmatteo.gagliardi@uniroma1.it (O.M.G.);; 2 University of Pittsburgh Medical Center, Pittsburgh, PA 15213, USA; 3 Department of Ophthalmology, Massachusetts Eye and Ear, Boston, MA 02114, USA; 4 Harvard Medical School, Boston, MA 02115, USA; 5 Eye Clinic, Department of Experimental and Clinical Medicine, Polytechnic University of Marche, 60126 Ancona, Italy

**Keywords:** artificial intelligence, deep learning, optical coherence tomography, intraretinal fluid, subretinal fluid, diabetic macular edema, neovascular age-related macular degeneration, macular telangiectasia, retinal fluid segmentation

## Abstract

**Objectives:** To validate a deep learning-based algorithm for automated detection, segmentation, and quantitative assessment of retinal fluid on optical coherence tomography (OCT) across multiple retinal diseases. **Methods:** In this retrospective validation study, spectral-domain OCT volume scans from eyes with neovascular age-related macular degeneration (nAMD), diabetic macular edema (DME), and macular telangiectasia type 2 (MacTel) were analyzed. Automated segmentation was performed using the fluid analyzer on Skopix by NetraMind Innovations, a deep learning-based framework trained to identify intraretinal fluid (IRF), subretinal fluid (SRF), pigment epithelial detachment (PED), and subretinal hyperreflective material (SHRM). Automated segmentations were reviewed and manually corrected by two masked experienced retina graders, with discrepancies adjudicated by a senior retina specialist. Scan-level detection performance was evaluated using accuracy, sensitivity, specificity, positive predictive value (PPV), negative predictive value (NPV), area under the receiver operating characteristic curve (AUC), and Gwet’s first-order agreement coefficient (AC1). Spatial segmentation agreement across all B-scans was assessed using the Dice coefficient. Quantitative agreement between automated and manually corrected fluid volumes was evaluated using correlation and Bland–Altman analyses. Inter-grader reliability was assessed using the intraclass correlation coefficient (ICC). **Results:** A total of 98 OCT volumes comprising 31 nAMD, 25 DME, and 42 MacTel eyes were included. In nAMD, IRF detection demonstrated a sensitivity of 81.8%, specificity of 83.0%, and AUC of 0.876, while SRF demonstrated a sensitivity of 81.4%, specificity of 96.7%, and AUC of 0.901. In DME, sensitivity, specificity, and AUC were 99.6%, 97.3%, and 0.989 for IRF and 98.4%, 96.4%, and 0.978 for SRF, respectively. In MacTel, IRF demonstrated a sensitivity of 87.3%, specificity of 95.1%, and AUC of 0.927, while SRF demonstrated a sensitivity of 86.8%, specificity of 98.4%, and AUC of 0.929. Segmentation agreement between automated and manually corrected annotations was high across disease groups, with Dice coefficients of 0.829 and 0.922 for IRF and SRF in nAMD, 0.858 and 0.941 in DME, and 0.908 and 0.97 in MacTel, respectively. Automated fluid volumes demonstrated strong correlations with manually corrected measurements, with minimal systematic bias on Bland–Altman analysis. **Conclusions:** The automated segmentation algorithm demonstrated strong validation performance for the detection and quantitative assessment of retinal fluid across nAMD, DME, and MacTel. High segmentation agreement, particularly for IRF and SRF, and strong volumetric agreement with expert-corrected measurements support the potential use of automated OCT fluid quantification in clinical practice and research.

## 1. Introduction

Intraretinal and subretinal fluid accumulation is a hallmark of several vision-threatening retinal diseases, including neovascular age-related macular degeneration (nAMD), diabetic macular edema (DME), central serous chorioretinopathy, and other causes of secondary choroidal neovascularization [1]. Intraretinal fluid (IRF), subretinal fluid (SRF), and pigment epithelial detachment (PED) are important structural biomarkers of disease activity, progression, and response to treatment [2]. The presence and evolution of these biomarkers influence treatment decisions, particularly in diseases managed with intravitreal anti-vascular endothelial growth factor (anti-VEGF) therapy [3]. Despite their clinical importance, reliable and reproducible quantitative assessment of retinal fluid remains challenging because of the considerable variability in fluid morphology, distribution, and burden across retinal diseases.

Optical coherence tomography (OCT) has become the imaging modality of choice for evaluating retinal fluid owing to its rapid acquisition and high-resolution cross-sectional visualization of retinal microstructure [4]. OCT enables the identification of intraretinal cystic spaces, subretinal fluid collections, and abnormalities of the retinal pigment epithelium that may not be evident on conventional fundus imaging. In routine clinical practice, however, fluid assessment remains predominantly qualitative and relies on clinician interpretation of sequential B-scans [5]. Although manual delineation of individual fluid compartments can provide a reference standard for quantitative analysis, such annotation is labor-intensive, time-consuming, and susceptible to interobserver variability, limiting its feasibility for large imaging datasets and high-volume clinical workflows [6].

Artificial intelligence, particularly deep learning (DL), has increasingly been applied to ophthalmic imaging for automated identification and segmentation of retinal layers and pathological biomarkers. DL-based models can process large OCT datasets rapidly and have shown promising performance for the identification of IRF, SRF, and other structural abnormalities [7]. Automated fluid quantification may improve reproducibility, reduce clinician workload, facilitate objective monitoring of treatment response, and provide standardized quantitative structural endpoints for clinical trials.

Despite these advances, generalizability remains an important challenge for DL-based imaging models [8]. Algorithms developed and tested on institution-specific or benchmark datasets may demonstrate reduced performance when applied to independent populations with different disease characteristics, fluid morphology, scan density, image quality, and acquisition protocols [9,10]. Validation using completely independent images, without additional model training or optimization, is therefore essential before automated segmentation systems can be considered for broad clinical or research implementation.

The heterogeneity of retinal fluid among different diseases provides an additional challenge. In nAMD, fluid may coexist with PED, subretinal hyperreflective material (SHRM), fibrosis, and irregular outer retinal architecture [11]. DME may contain numerous cystoid spaces distributed throughout the retina with substantially greater fluid volumes [12]. In contrast, MacTel is characterized by inner and outer retinal cavitations that may resemble exudative fluid despite a different pathophysiological mechanism [13]. Evaluation across these phenotypes therefore provides a rigorous assessment of model robustness.

In the present study, we performed an independent validation of a deep learning-based automated OCT algorithm for the identification, segmentation, and quantitative assessment of retinal fluid. Performance was evaluated in nAMD, DME, and MacTel using expert-corrected segmentations as the reference standard. Detection, spatial segmentation, and quantitative volumetric performance were assessed separately for each disease and fluid compartment to determine the accuracy, reproducibility, and potential clinical applicability of the automated approach.

## 2. Materials and Methods

### 2.1. Study Design and Dataset

This retrospective single-center, single-device cross-disease validation study was conducted in the Department of Ophthalmology at the University of Pittsburgh Medical Center in accordance with the tenets of the Declaration of Helsinki and was approved by the Institutional Review Board. OCT volume scans from eyes with nAMD, DME, and MacTel were analyzed for validation of the automated segmentation algorithm. To avoid inter-eye correlation bias, only one eye per participant was included. Archived OCT volumes were retrieved from the institutional imaging database. Investigators had access only to limited clinical information, including the diagnosis associated with each OCT volume. No direct identifiers were available to the study team during or after data collection.

Spectral-domain OCT volume scans were obtained using the Heidelberg Spectralis system (Heidelberg Engineering, Heidelberg, Germany). Only scans with a quality score of 25 or higher were included. All volumes covered a 6 × 6 mm area centered on the fovea, with individual B-scans consisting of 512 × 496 pixels. Candidate OCT volumes were screened for inclusion in the independent validation dataset. Volumes previously included in the model training dataset were excluded to ensure complete separation between training and validation data. Scans acquired using Cirrus OCT and those not meeting the predefined image-quality threshold were also excluded. One eye per participant was included. Different raster acquisition protocols were used according to disease group, comprising 49 to 97 B-scans per volume (49 B-scans per volume for DME, 61 B-scans per volume for AMD, and 97 B-scans per volume for MacTel). These differences reflected the standard disease-specific imaging protocols established at our institution and were independent of the study design. A total of 98 OCT volumes were included, comprising 31 nAMD, 25 DME, and 42 MacTel volumes. IRF and SRF were evaluated in all three disease cohorts. IRF and SRF in MacTel corresponded to the inner retinal cavitation and outer retinal cavitation. Although the algorithm is capable of segmenting and quantifying PED and SHRM, these biomarkers were not included in the present validation analysis because they could not be evaluated consistently across all three disease cohorts.

### 2.2. Automated Fluid Segmentation

Automated retinal fluid segmentation was performed using the fluid analyzer on Skopix, a proprietary deep learning-based framework developed by NetraMind Innovations (Pittsburgh, PA, USA) [14]. The framework consists of a Pix2Pix generative adversarial network with a Residual U–Net generator designed to perform pixel-wise segmentation of retinal pathological features on individual OCT B-scans.

The model was trained using a multi-device dataset comprising approximately 1200 Heidelberg Spectralis OCT volumes and 3000 Cirrus HD–OCT volumes obtained from healthy eyes and eyes with age-related macular degeneration, diabetic retinopathy, and central serous chorioretinopathy. Training was performed for 100 epochs using the Adam optimizer and a combined segmentation and adversarial loss function. Standard data augmentation techniques, including image flipping, translation, scaling, and intensity perturbation, were used during training. The trained network generated segmentation masks for IRF, SRF, and PED.

The present study constituted a newly assembled, independent dataset that was completely held out from model development. None of the validation scans were used for algorithm training, and no additional training, fine-tuning, or optimization was performed using the validation dataset. For the MacTel cohort, hyporeflective cavitations were classified according to their anatomical location for algorithm validation. Spaces identified as IRF were considered inner retinal cavitations, while those identified as SRF were considered outer retinal cavitations. These were operational anatomical labels rather than indicators of exudative fluid, used to assess the algorithm’s potential for location-based cavitation quantification in future MacTel studies.

### 2.3. Reference Standard and Manual Correction

Following automated segmentation, two masked experienced retina graders (NH and GG) independently reviewed the OCT volumes and manually corrected segmented fluid spaces where necessary using a freehand annotation tool. Fluid boundaries were modified or redrawn to accurately delineate IRF and SRF. The manually corrected segmentations served as the reference standard. Inter-grader agreement was assessed, and discrepancies between graders were reviewed with the senior author (J.C.). The final consensus segmentation was used as the reference standard for subsequent analyses.

### 2.4. Detection and Quantitative Evaluation

Algorithm performance was evaluated at the B-scan detection, spatial segmentation, and volumetric levels. For detection analysis, each B-scan was classified as positive or negative for the presence of each retinal biomarker according to the manually corrected reference standard. IRF and SRF detection were evaluated in nAMD, DME, and MacTel. For spatial segmentation analysis, Dice coefficients were calculated across all B-scans with the corresponding retinal biomarker. This approach was applied consistently across disease groups and retinal compartments.

For quantitative assessment, fluid volumes were calculated separately for each compartment by summing segmented areas across all B-scans within each OCT volume and converting these measurements to cubic millimeters using the physical voxel dimensions. Automated volumes were compared with the corresponding manually corrected reference measurements.

### 2.5. Statistical Analysis

Statistical analyses were performed using Python version 3.10 (Python Software Foundation, Wilmington, DE, USA) with appropriate scientific computing and statistical libraries. Fluid volumes were expressed as mean ± standard deviation. Inter-grader reliability between the two masked graders was assessed using the intraclass correlation coefficient (ICC). Agreement between automated and manual identification of the presence or absence of each retinal biomarker on individual B-scans was assessed using Gwet’s first-order agreement coefficient (AC1).

Scan-level detection performance was evaluated using accuracy, sensitivity, specificity, and area under the receiver operating characteristic curve (AUC) with 95% confidence intervals. Spatial agreement between automated and manually corrected segmentations was evaluated using the Dice coefficient calculated across all B-scans. Pearson correlation coefficients were used to evaluate the relationship between automated and manually corrected fluid volumes. Bland–Altman analysis was used to assess systematic measurement bias and 95% limits of agreement [15]. Proportional bias was assessed by evaluating the relationship between measurement differences and mean measurements. Root mean square error (RMSE) and mean absolute error (MAE) were also calculated. All statistical analyses were performed separately for each disease group and retinal biomarker. Statistical significance was defined as a two-sided *p* value < 0.05.

OpenAI ChatGPT (GPT−5.6, San Francisco, CA, USA) was used solely for language editing, including the improvement of grammar, clarity, and readability. All AI-assisted text was subsequently reviewed and verified by the authors.

## 3. Results

### 3.1. Study Cohort

**Figure 1:**
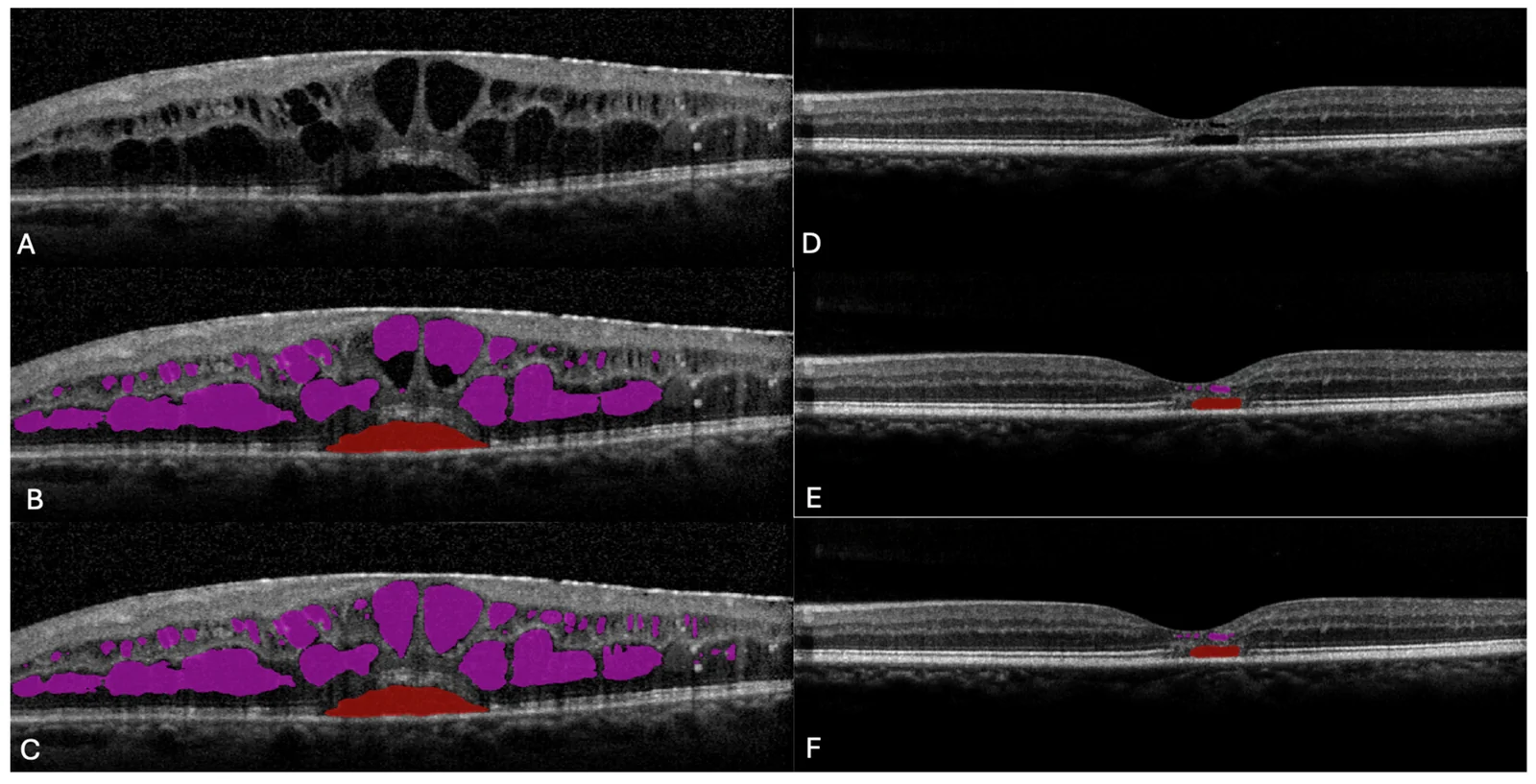
Representative B-scan images and automated and manually corrected segmentation of retinal fluid. Representative OCT B-scans from (**A**–**C**) DME and (**D**–**F**) MacTel. The first row (**A**,**D**) shows the original unsegmented B-scan images; the second column (**B**,**E**) shows automated segmentation; and the third column (**C**,**F**) shows the corresponding manually corrected segmentation. IRF is highlighted in pink and SRF in red. At higher fluid volumes, greater discrepancies were observed between automated and manual segmentations, resulting in reduced quantitative agreement and increasing underestimation by the automated algorithm.

A total of 98 OCT volumes from 98 eyes were included, comprising 31 nAMD, 25 DME, and 42 MacTel eyes (Figure 1). This corresponded to 1975 individual B-scans in the nAMD cohort, 1225 B-scans in the DME cohort, and 4074 B-scans in the MacTel cohort. Mean manually corrected IRF volume was 0.255 ± 0.847 mm^3^ in nAMD, 3.110 ± 3.776 mm^3^ in DME, and 0.292 ± 0.336 mm^3^ in MacTel. Mean SRF volume was 0.832 ± 2.367 mm^3^ in nAMD, 0.218 ± 0.415 mm^3^ in DME, and 0.264 ± 0.494 mm^3^ in MacTel (Table 1). Inter-grader reliability was excellent for SRF, with an ICC of 0.995 (95% CI, 0.972–1.000). For IRF, the ICC point estimate was 0.831; however, the wide 95% CI (0.229–0.977) indicated considerable uncertainty in the precision of this estimate.

For MacTel, IRF and SRF are operational algorithmic labels based on anatomical location and correspond to inner retinal and outer retinal cavitations, respectively; these terms do not imply exudative fluid.

### 3.2. Automated Detection, Segmentation, and Volumetric Performance

**Figure 2:**
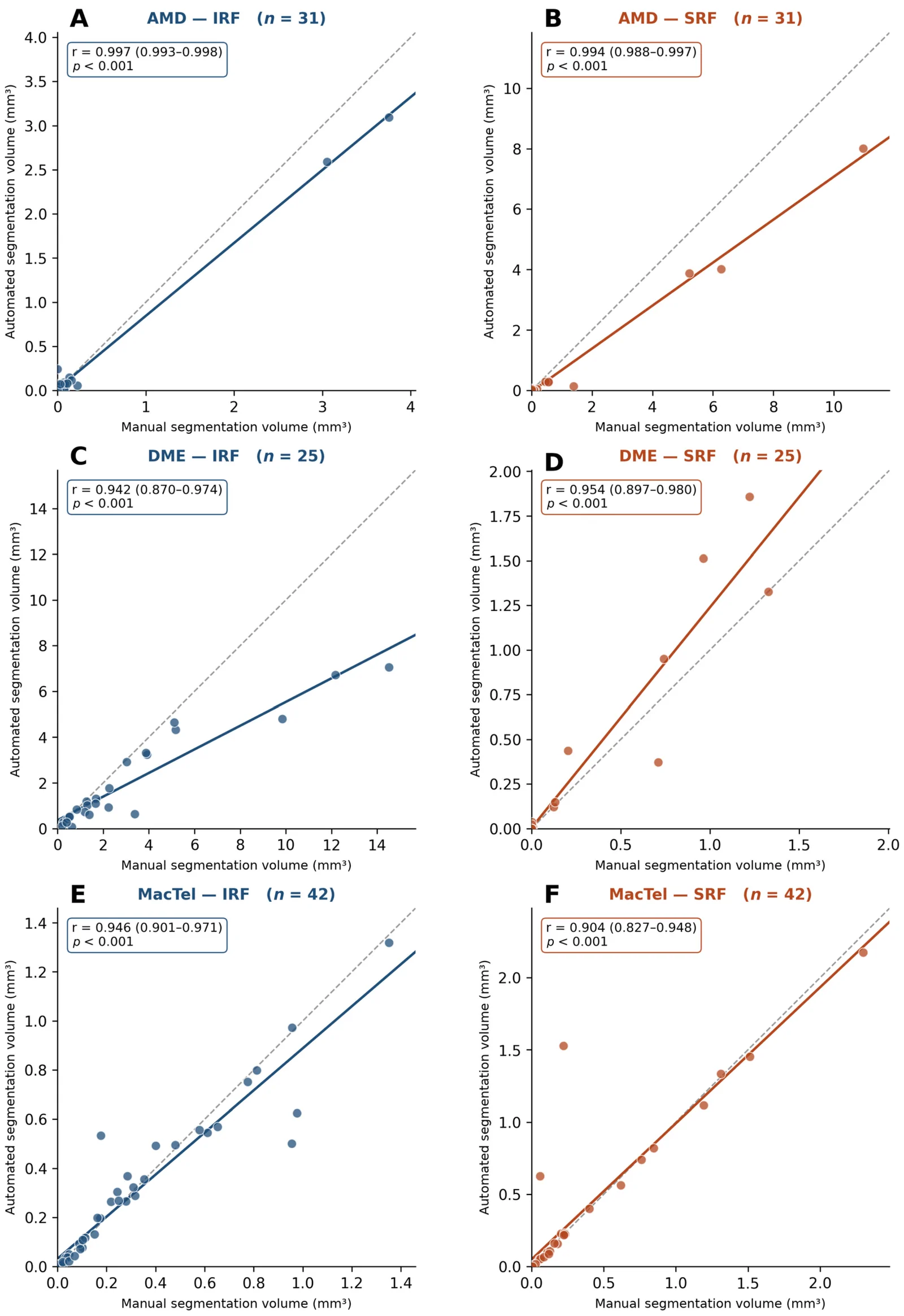
Scatterplots demonstrate the relationship between predicted and ground-truth (GT) fluid volumes for (**A**) AMD–IRF, (**B**) AMD–SRF, (**C**) DME–IRF, (**D**) DME–SRF, (**E**) MacTel–IRF, and (**F**) MacTel–SRF. The solid line represents the linear regression fit, and the dashed line represents the line of identity (predicted = ground truth). Strong correlations between predicted and GT fluid volumes were observed across all disease and fluid categories (Pearson *r* = 0.904–0.997; all *p* < 0.001). Deviation of the regression line from the line of identity at higher fluid volumes, particularly for AMD–SRF and DME–IRF, suggests increasing underestimation at greater fluid volumes. Insets in each panel provide a magnified view of the 0–1.0 mm^3^ region to facilitate visualization of agreement at lower fluid volumes.

Among 1975 nAMD B-scans, IRF detection demonstrated an accuracy of 82.9%, sensitivity of 81.8%, specificity of 83.0%, and AUC of 0.876, with a Gwet’s AC1 of 0.779 and Dice coefficient of 0.829. SRF detection demonstrated an accuracy of 95.0%, sensitivity of 81.4%, specificity of 96.7%, and AUC of 0.901, with a Gwet’s AC1 of 0.938 and Dice coefficient of 0.922 (Table 2). Automated and manually corrected volumetric measurements demonstrated strong correlations for IRF (Pearson *r* = 0.997) and SRF (*r* = 0.994) (Figure 2A,B).

Among 1225 DME B-scans, IRF detection demonstrated an accuracy of 98.7%, sensitivity of 99.6%, specificity of 97.3%, and AUC of 0.989, with a Gwet’s AC1 of 0.975 and Dice coefficient of 0.858. SRF detection demonstrated an accuracy of 96.6%, sensitivity of 98.4%, specificity of 96.4%, and AUC of 0.978, with a Gwet’s AC1 of 0.957 and Dice coefficient of 0.941 (Table 2). Automated and manually corrected fluid volumes demonstrated strong correlations for both IRF (Pearson *r* = 0.942) and SRF (*r* = 0.954) (Figure 2C,D).

Among MacTel volumes, IRF detection demonstrated an accuracy of 93.9%, sensitivity of 87.3%, specificity of 95.1%, and AUC of 0.927, with a Gwet’s AC1 of 0.918 and Dice coefficient of 0.908. SRF detection demonstrated an accuracy of 97.6%, sensitivity of 86.8%, specificity of 98.4%, and AUC of 0.929, with a Gwet’s AC1 of 0.972 and Dice coefficient of 0.97 (Table 2). Automated and manually corrected fluid volumes demonstrated strong correlations for both IRF (Pearson *r* = 0.946) and SRF (*r* = 0.904) (Figure 2E,F).

**Figure 3:**
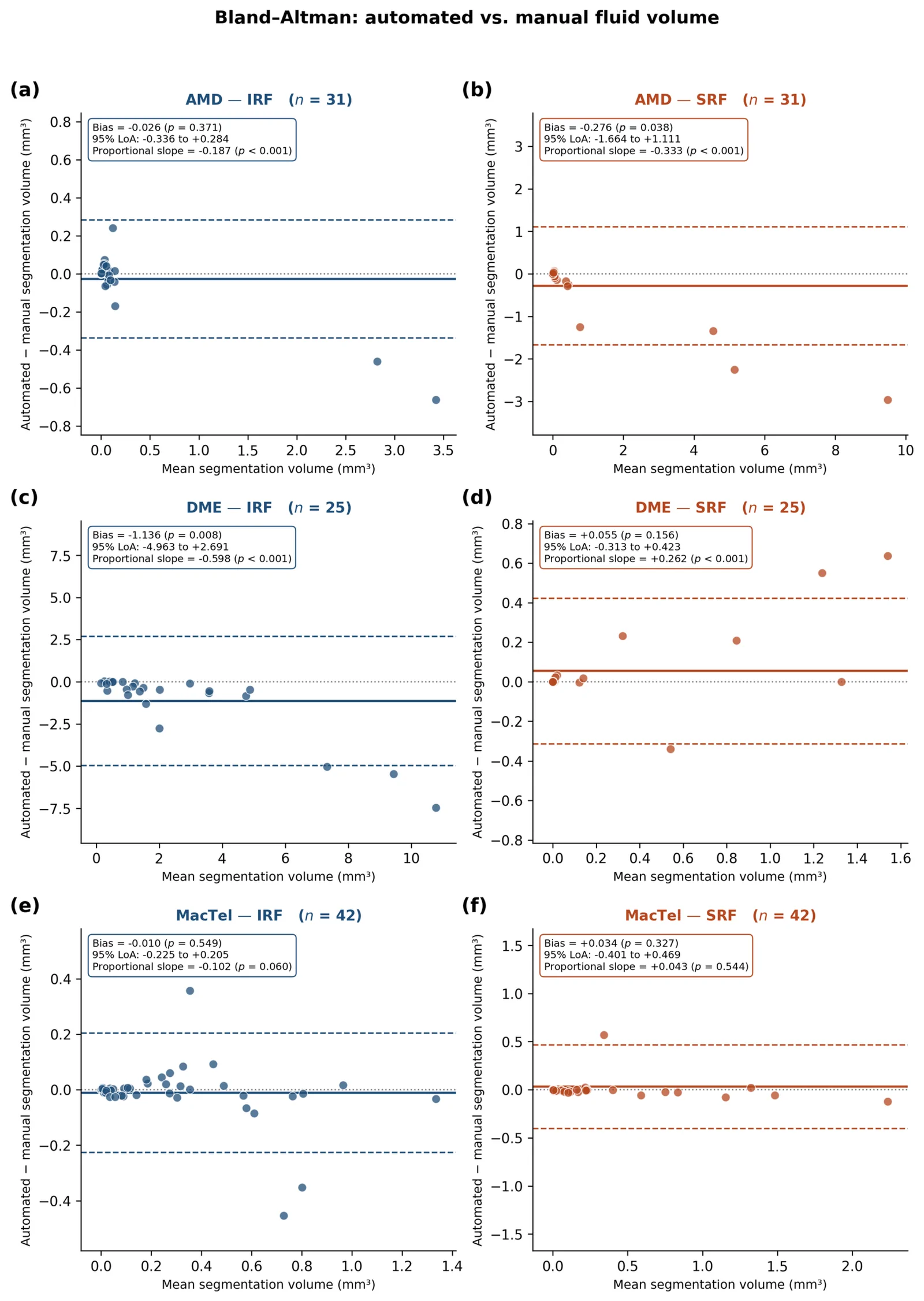
Bland–Altman analysis of agreement between automated and ground-truth retinal fluid volumes for (**A**) AMD–IRF (**B**) AMD–SRF (**C**) DME–IRF (**D**) DME–SRF (**E**) MacTel–IRF, and (**F**) MacTel–SRF. The solid horizontal line represents the mean bias and dashed lines represent the 95% limits of agreement (mean bias ± 1.96 SD). AMD–IRF and AMD–SRF showed relatively small mean biases but significant proportional biases, with increasing underestimation at larger fluid volumes. DME–IRF demonstrated the greatest systematic underestimation and wide limits of agreement, particularly at higher fluid volumes, whereas DME–SRF showed minimal overall bias but significant proportional bias. MacTel–IRF and MacTel–SRF demonstrated the best agreement, with minimal mean bias, relatively narrow limits of agreement, and no statistically significant proportional bias.

Bland–Altman analysis demonstrated variable volumetric agreement across disease and fluid categories (Figure 3). MacTel showed the closest agreement, with minimal mean bias for both IRF (−0.010 mm^3^) and SRF (0.034 mm^3^) and no significant proportional bias. DME–SRF similarly demonstrated minimal mean bias (0.055 mm^3^), although proportional bias was present. In contrast, DME–IRF showed the greatest systematic underestimation, with a mean bias of −1.136 mm^3^ and wider limits of agreement, particularly at higher fluid volumes. AMD–IRF and AMD–SRF also demonstrated increasing underestimation with greater fluid burden. Overall, despite strong volumetric correlations, Bland–Altman analysis indicated reduced agreement at higher fluid volumes, most prominently for DME–IRF and AMD–SRF.

## 4. Discussion

Our study demonstrates strong validation performance of a deep learning-based algorithm for automated detection, segmentation, and quantitative assessment of retinal fluid across three distinct retinal diseases: nAMD, DME, and MacTel. The algorithm demonstrated high spatial agreement for IRF and SRF, strong scan-level detection performance, and close correlation between automated and manually corrected fluid volumes. Importantly, the model was evaluated on an independent dataset without additional training or fine-tuning, providing an assessment of its performance outside its development dataset.

Manual reference grading demonstrated high reproducibility. Inter-grader reliability was excellent for SRF (ICC, 0.995; 95% CI, 0.972–1.000) and good for IRF (ICC, 0.831; 95% CI, 0.229–0.977). The particularly high agreement for SRF is consistent with the comparatively well-defined anatomical boundaries of this compartment. The wider confidence interval for IRF reflects greater variability in the delineation of complex intraretinal cystic spaces. Nevertheless, the observed inter-grader reliability supports the use of expert-corrected annotations as the reference standard for algorithm validation.

The scan-level detection performance observed in the present study compares favorably with previously reported automated fluid detection algorithms. In the Age-Related Eye Disease Study 2 follow-on study, an automated OCT algorithm demonstrated sensitivities of 76.3% and 94.0% and specificities of 92.2% and 85.7% for IRF and SRF, respectively [16]. In our nAMD cohort, IRF sensitivity and specificity were 81.8% and 83.0%, while SRF sensitivity and specificity were 81.4% and 96.7%, respectively. Thus, our model demonstrated slightly higher IRF sensitivity and substantially higher SRF specificity, although direct comparisons should be interpreted cautiously because of differences in datasets, disease characteristics, and reference standards.

The spatial segmentation performance observed in our study is also broadly consistent with contemporary DL-based fluid segmentation studies. Karn et al. evaluated a multiscale attention-based U–Net architecture using the RETOUCH dataset and reported F1 scores of 0.81 for IRF and 0.95 for SRF on Spectralis images [17]. Wilson et al. reported model-grader Dice coefficients of 0.43 for IRF and 0.78 for SRF across their AMD and DME dataset. In comparison, Dice coefficients in our nAMD cohort were 0.829 for IRF and 0.922 for SRF [18]. Although these values compare favorably with previous reports, differences in datasets, disease composition, definitions, and analytical approaches preclude direct conclusions regarding superiority.

The algorithm demonstrated particularly strong performance in DME, with IRF and SRF detection achieving accuracies of 98.7% and 96.6%, sensitivities of 99.6% and 98.4%, and specificities of 97.3% and 96.4%, respectively. Agreement between automated and manually corrected assessments was similarly high, with Gwet’s AC1 values of 0.975 for IRF and 0.957 for SRF and Dice coefficients of 0.858 and 0.941, respectively. Automated fluid volumes also demonstrated strong correlations with manually corrected measurements for both IRF (Pearson *r* = 0.942) and SRF (*r* = 0.954). These findings compare favorably with a previous multicenter study of 303 eyes with DME, in which AI-based OCT analysis was considered clinically accurate for IRF and SRF quantification in 95.4% and 94.7% of eyes, respectively [19]. For SRF, the previous algorithm demonstrated a sensitivity of 89.7%, specificity of 97.0%, and Gwet’s AC1 of 0.95 (95% CI, 0.92–0.98). In comparison, our algorithm demonstrated higher SRF sensitivity (98.3%), comparable specificity (96.4%), and similarly high agreement (Gwet’s AC1 = 0.957).

Despite strong correlations between automated and manually corrected fluid volumes across all cohorts, volumetric agreement was reduced at higher fluid burdens. This was most apparent for DME–IRF and AMD–SRF, where the regression plots demonstrated increasing deviation from the line of identity and Bland–Altman analysis showed greater underestimation with increasing fluid volume. Thus, the high Pearson correlation coefficients reflect strong association between automated and reference measurements but should not be interpreted as equivalent to absolute agreement, particularly in eyes with large fluid volumes.

MacTel provided an important and distinct test of algorithm generalizability. The retinal cavitations characteristic of MacTel differ structurally and pathophysiologically from the exudative fluid commonly encountered in nAMD and DME. Nevertheless, the algorithm maintained strong performance. Dice coefficients were 0.908 for IRF and 0.970 for SRF, while Gwet’s AC1 values were 0.918 and 0.972, respectively. These findings demonstrate strong agreement between automated and manually corrected assessments across the MacTel dataset. Quantitative agreement in MacTel was also strong. Automated and manually corrected IRF volumes demonstrated a Pearson correlation coefficient of 0.946, while SRF volumes demonstrated a correlation coefficient of 0.904. Neither compartment demonstrated statistically significant proportional bias. Thus, strong spatial agreement was accompanied by close whole-volume quantitative agreement.

The MacTel findings nevertheless require disease-specific interpretation. Hyporeflective cavitations in MacTel do not necessarily represent active vascular leakage, and in clinical and research assessments these spaces are often considered collectively rather than interpreted as conventional exudative IRF or SRF [13]. Therefore, automated classification of these spaces as IRF or SRF in the present study primarily reflects their anatomical location and structural appearance on OCT and should not be interpreted as evidence of exudative disease activity. However, the ability of the algorithm to distinguish and quantify cavitations according to their retinal location may provide additional information beyond their combined assessment. It may be hypothesized that cavitations occurring in different retinal compartments represent distinct structural or pathophysiological processes and may consequently have different associations with disease progression and visual prognosis. Longitudinal studies correlating layer-specific cavitation volumes with retinal structural changes and visual function are warranted to determine whether such compartment-specific quantification has prognostic significance in MacTel.

The validation design is particularly important for the translation of AI-based algorithms into clinical practice. DL models frequently demonstrate strong performance on internal test sets but may perform differently when applied to independent populations because of differences in disease severity, imaging quality, acquisition protocols, and retinal morphology. In the present study, no additional training or fine-tuning was performed using the validation dataset. Furthermore, the OCT acquisition protocols varied from 49 to 97 B-scans per volume. The maintenance of strong performance under these conditions supports the robustness of the algorithm outside its original development environment.

The present study has several limitations. First, this was a retrospective study conducted at a single institution. Second, all validation images were acquired using the Heidelberg Spectralis platform. Although the algorithm was developed using images from multiple OCT devices, independent validation on additional imaging platforms is warranted. Third, the number of eyes within individual disease groups was relatively limited despite the large number of individual B-scans analyzed. Fourth, different raster acquisition protocols were used among the disease groups, which may influence volumetric measurements but also reflect variability encountered in clinical imaging practice. Fifth, expert manual correction was used as the reference standard, and some subjectivity remains unavoidable, particularly for small fluid spaces. The good-to-excellent inter-grader reliability observed for IRF and SRF partly mitigates this limitation, and disagreements were adjudicated by a senior retina specialist. Expert correction of automated segmentations, rather than independent de novo annotation, was used to establish the reference standard because fully manual pixel-level segmentation across the large number of B-scans was resource-intensive. Although this correction-based approach has been used in our previous work [14] and all segmentations were independently reviewed by experienced retina graders with adjudication of disagreements, prior exposure to the automated segmentation may introduce anchoring or automation bias and potentially increase apparent agreement. Future validation using independently generated de novo annotations would provide a more stringent assessment of segmentation performance.

Finally, the present study evaluated cross-sectional segmentation and quantitative performance. Future longitudinal studies should determine whether automated changes in fluid volume accurately reflect treatment response, disease recurrence, and functional outcomes.

In conclusion, this validation study demonstrates strong performance of a deep learning-based algorithm for automated detection, segmentation, and quantitative assessment of retinal fluid across nAMD, DME, and MacTel. High Dice coefficients, strong categorical agreement, and close volumetric agreement with expert-corrected measurements support the robustness of the automated approach across substantially different retinal phenotypes. Further multicenter, cross-device, and longitudinal validation will help establish the role of automated fluid quantification in clinical practice and clinical trials.

## Figures and Tables

**Table 1:** Comparison of automated and manually corrected fluid volumes across retinal disease cohorts.

Metric	AMD	AMD	DME	DME	MacTel	MacTel
Fluid	IRF	SRF	IRF	SRF	IRF	SRF
Number of Eyes	31	31	25	25	42	42
Automated Segmentation Volume (Mean ± SD), mm^3^	0.229 ± 0.702	0.555 ± 1.693	1.974 ± 2.073	0.273 ± 0.537	0.282 ± 0.304	0.298 ± 0.514
Manually Corrected Volume (mean ± SD), mm^3^	0.255 ± 0.847	0.832 ± 2.367	3.110 ± 3.776	0.218 ± 0.415	0.292 ± 0.336	0.264 ± 0.494
Mean Difference (Mean ± SD), mm^3^	−0.026 ± 0.158	−0.276 ± 0.701	−1.136 ± 1.953	+0.055 ± 0.188	−0.01 ± 0.11	+0.034 ± 0.222
Pearson r (95% CI)	0.997 (0.993–0.998)	0.994 (0.988–0.997)	0.942 (0.870–0.974)	0.954 (0.897–0.980)	0.946 (0.901–0.971)	0.904 (0.827–0.948)
RMSE (mm^3^) (95% CI)	0.223 (0.043–0.354)	1.257 (0.434–1.823)	2.225 (0.787–3.257)	0.226 (0.081–0.336)	0.112 (0.036–0.163)	0.236 (0.024–0.396)
MAE (mm^3^) (95% CI)	0.118 (0.036–0.233)	0.801 (0.260–1.415)	1.139 (0.467–1.994)	0.115 (0.031–0.219)	0.051 (0.025–0.083)	0.067 (0.013–0.151)

AMD: Age-related macular degeneration; DME: diabetic macular edema; MacTel: macular telangiectasia; IRF: intraretinal fluid; SRF: subretinal fluid; RMSE: root mean square error; MAE: mean absolute error.

**Table 2:** Scan-level detection and spatial segmentation performance of the automated algorithm across retinal disease cohorts.

Metric	AMD	AMD	DME	DME	MacTel	MacTel
Fluid	IRF	SRF	IRF	SRF	IRF	SRF
No. of Eyes	31	31	25	25	42	42
Gwet’s AC1 (95%CI)	0.779 (0.754–0.804)	0.938 (0.926–0.951)	0.975 (0.963–0.987)	0.957 (0.944–0.970)	0.918 (0.907–0.928)	0.972 (0.967–0.978)
Dice Coefficient (95%CI)	0.829 (0.813–0.844)	0.922 (0.911–0.932)	0.858 (0.846–0.869)	0.941 (0.929–0.952)	0.908 (0.9–0.916)	0.97 (0.965–0.975)
Sensitivity (95%CI)	0.818 (0.736–0.879)	0.814 (0.756–0.861)	0.996 (0.988–0.999)	0.984 (0.943–0.996)	0.873 (0.843–0.897)	0.868 (0.822–0.903)
Specificity (95%CI)	0.830 (0.812–0.846)	0.967 (0.957–0.974)	0.973 (0.955–0.984)	0.964 (0.951–0.973)	0.950 (0.943–0.957)	0.983 (0.979–0.987)
Accuracy (95%CI)	0.829 (0.812–0.845)	0.950 (0.940–0.959)	0.987 (0.979–0.992)	0.966 (0.954–0.975)	0.939 (0.932–0.946)	0.976 (0.971–0.980)
AUC (95%CI)	0.876 (0.832–0.913)	0.901 (0.872–0.927)	0.989 (0.981–0.994)	0.978 (0.964–0.989)	0.927 (0.912–0.940)	0.929 (0.907–0.948)

AMD: Age-related macular degeneration; DME: diabetic macular edema; MacTel: macular telangiectasia; IRF: intraretinal fluid; SRF: subretinal fluid; AUC: area under the curve.

## Data Availability

The data presented in this study are available on request from the corresponding author due to patient privacy and institutional regulations.
